# SAM Domain-Based Protein Oligomerization Observed by Live-Cell Fluorescence Fluctuation Spectroscopy

**DOI:** 10.1371/journal.pone.0001931

**Published:** 2008-04-23

**Authors:** Brian D. Slaughter, Joseph M. Huff, Winfried Wiegraebe, Joel W. Schwartz, Rong Li

**Affiliations:** The Stowers Institute for Medical Research, Kansas City, Missouri, United States of America; Ordway Research Institute, United States of America

## Abstract

Sterile-alpha-motif (SAM) domains are common protein interaction motifs observed in organisms as diverse as yeast and human. They play a role in protein homo- and hetero-interactions in processes ranging from signal transduction to RNA binding. In addition, mutations in SAM domain and SAM-mediated oligomers have been linked to several diseases. To date, the observation of heterogeneous SAM-mediated oligomers *in vivo* has been elusive, which represents a common challenge in dissecting cellular biochemistry in live-cell systems. In this study, we report the oligomerization and binding stoichiometry of high-order, multi-component complexes of (SAM) domain proteins Ste11 and Ste50 in live yeast cells using fluorescence fluctuation methods. Fluorescence cross-correlation spectroscopy (FCCS) and 1-dimensional photon counting histogram (1dPCH) confirm the SAM-mediated interaction and oligomerization of Ste11 and Ste50. Two-dimensional PCH (2dPCH), with endogenously expressed proteins tagged with GFP or mCherry, uniquely indicates that Ste11 and Ste50 form a heterogeneous complex in the yeast cytosol comprised of a dimer of Ste11 and a monomer of Ste50. In addition, Ste50 also exists as a high order oligomer that does not interact with Ste11, and the size of this oligomer decreases in response to signals that activate the MAP kinase cascade. Surprisingly, a SAM domain mutant of Ste50 disrupted not only the Ste50 oligomers but also Ste11 dimerization. These results establish an *in vivo* model of Ste50 and Ste11 homo- and hetero-oligomerization and highlight the usefulness of 2dPCH for quantitative dissection of complex molecular interactions in genetic model organisms such as yeast.

## Introduction

Determining the state of protein complex formation is critical for understanding many signaling and structural pathways. Often protein interactions are mediated through conserved domains, such as the well-studied Src homology 3 (SH3) and PDZ domains [1]. The Sterile-alpha-motif (SAM) domain is another commonly occurring motif, facilitating diverse interactions including protein homo-dimerization, hetero-dimerization, and even RNA binding [2], [3]. Defects in the SAM domains of proteins have been observed in a number of human diseases [2], [4]–[8]. Notably, chromosomal translocation of the ETS family transcriptional regulator TEL (translocation Ets leukemia), a SAM-domain containing protein, has been frequently linked to human leukemias and it is thought that the diseases arise because SAM-mediated oligomerization constitutively activates mitogenic proteins [6], [9]–[11]. The importance of this domain has led to numerous studies determining the structure and stoichiometry of SAM-domain complexes [2]. Although *in vitro* SAM domains are capable of forming both homo- and hetero-oligomers, it remains unclear how SAM domains mediate protein interactions under *in vivo* settings, where most proteins are expressed at levels much lower than those often used in biochemical and structural analyses.

In yeast, Ste11 and Ste50 are SAM domain-containing signaling proteins involved in multiple morphogenetic pathways, including mating, invasive growth, and high-osmolarity response [12]–[14]. The interaction of these proteins through their SAM domains is thought to play a role in the delivery of Ste11, a MAP kinase kinase kinase, to the cell cortex to activate MAP kinase in response to environmental signals [15], [16]. Several groups have employed structural and biochemical methods to examine homo and hetero interactions of purified SAM domains of Ste11 and Ste50 in solution [17]–[21]. A consensus of their study is that Ste11 SAM domains form tight homodimers or high-order oligomers, whereas the Ste50 SAM domain, with a slightly different sequence, is largely monomeric in solution but can mediate strong heterodimerization with the Ste11 SAM domain [17], [18], [20], [21]. It is unknown, however, how these domains might be engaged in hetero or homo-oligomer formation *in vivo*, in the presence of the full length proteins expressed at their endogenous levels and in the presence of additional interacting partners.

**Figure 1:**
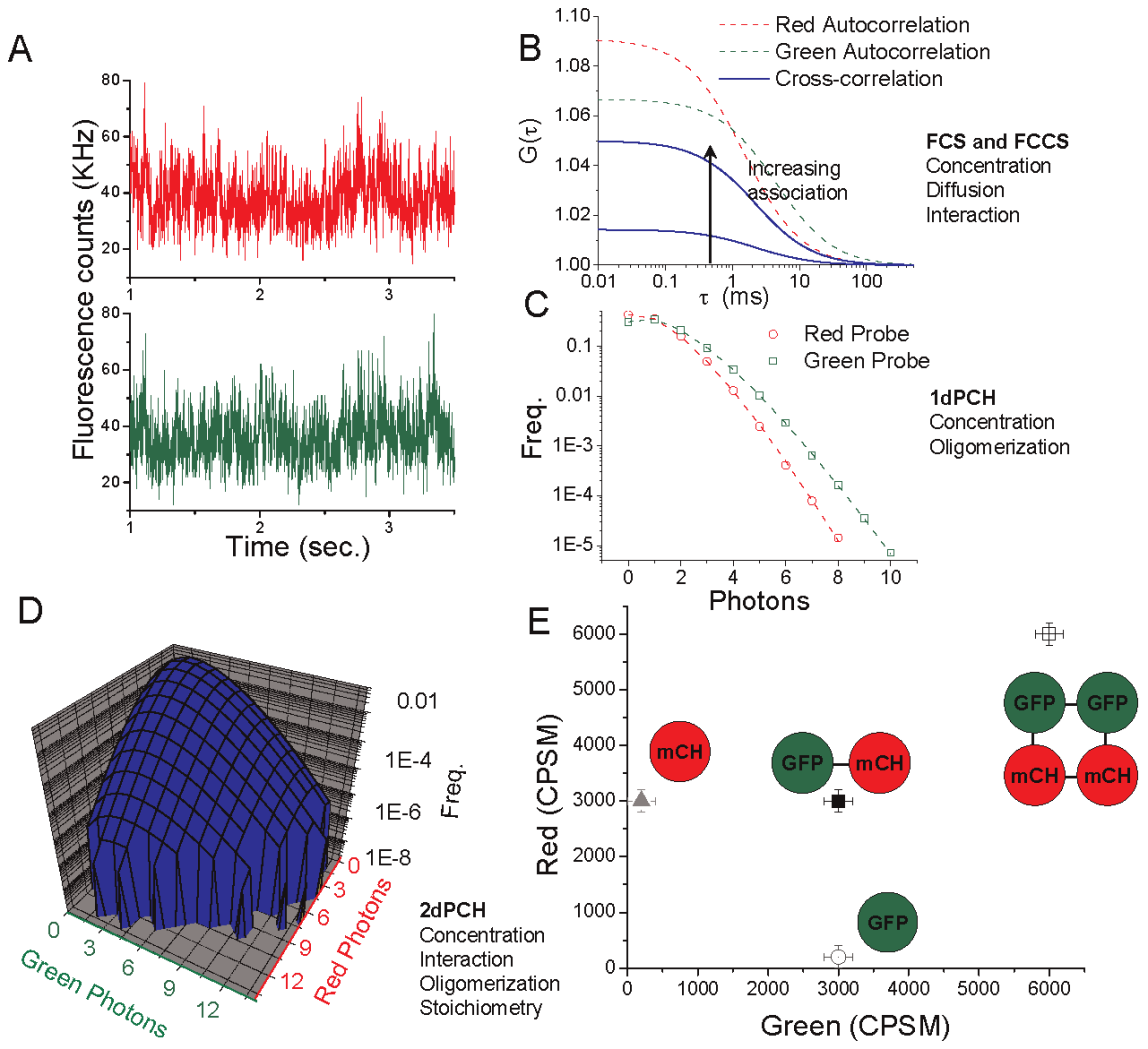
Fluctuation data can probe protein-protein interactions. A. Example traces of fluctuation data for dual-color experiments. B. Data can be analyzed by correlation analysis to examine concentration, diffusion, and co-diffusion of red and green particles. C. 1dPCH examines the distribution of photon events per time interval, and reports concentration and ‘brightness’, or oligomeric status. D. 2dPCH reports simultaneously concentration, interaction, oligomerization, and binding stoichiometry of heterogeneous complexes. An example two-dimensional PCH histogram is shown, with frequency versus number of green photons and number of red photons per time bin. E. Example, two-dimensional plot of a fit of modeled 2dPCH data. If a monomer red or green probe has a brightness of 3000 CPSM, for example, the plot demonstrates points one would expect to find values for with non-interacting monomeric species, or interacting monomeric species, or interacting dimeric species, as labeled.

Emerging fluorescence-based technologies probe *in vivo* binding equilibrium and stoichiometry of protein complexes. Fluorescence correlation spectroscopy (FCS) and fluorescence cross-correlation spectroscopy (FCCS) [22]–[26] are fluctuation techniques that analyze protein mobility, concentration, and protein-protein association (Figure 1A, 1B), and have recently been applied to live yeast cells expressing autofluorescent proteins (AFP) at the endogenous levels [27], [28]. While FCCS measures co-diffusion of two particles with different fluorescent tags, extraction of binding stoichiometry is not easily accomplished with this technique. The photon counting histogram (PCH) and similar techniques, such as fluorescence intensity distribution analysis (FIDA), are fluctuation techniques designed to analyze the oligomeric status of fluorescent species and have been applied to both *in vitro* and *in vivo* systems. These techniques determine the state of molecular homo-oligomerization [29]–[34] (Figure 1C); however, PCH does not resolve stoichiometry of heterogeneous complexes that result from dynamic protein interactions between different molecular species.

Two-dimensional PCH (2dPCH), where two different proteins are tagged with spectrally distinct probes, is a recently developed technique that can be used for simultaneous measurement of stoichiometry and interaction [35], [36]. In 2dPCH, a two-dimensional histogram of fluorescence counts in red and green channels is generated (Figure 1A, 1D) from fluctuation data. The surface of the two-dimensional histogram can be fit to yield a two-dimensional map of the brightness in each channel of diffusing species. For example, a hypothetical monomeric green probe diffusing alone with a brightness of 3000 counts per second per molecule would surface in the plot with a brightness in the green channel without a contribution in the red channel (Figure 1E). A red probe diffusing with no green particle would likewise only have a contribution in the red channel. However, if monomeric red and green probes are co-diffusing, the corresponding two-dimensional histogram would be best fit by a diffusing species with brightness contributions in both channels. In this way, both co-diffusion and stoichiometry of heterogeneous complexes may be observed. As a new technique, its *in vivo* applications have been limited to this point [35]. However, the combination of GFP with the improved red AFP, mCherry [37], [38], and the ease of introducing these tags to chromosomal loci through homologous recombination in yeast make it feasible to apply 2dPCH to assess high-order heterogeneous protein complexes in live yeast cells.

In this study, we apply 2dPCH, FCCS, and 1d PCH in live budding yeast cells to examine the interaction between SAM domain-containing proteins Ste11 and Ste50 and the effect of mutations in Ste50's SAM domain on homotypic and heterotypic protein interactions. The data allows establishment of a dynamic model depicting homo and hetero-oligomeric complex formation among these SAM domain proteins, and represents the first application of 2dPCH to extract stoichiometry of high order heterogeneous complexes using endogenously expressed proteins in live cells.

## Results

**Figure 2:**
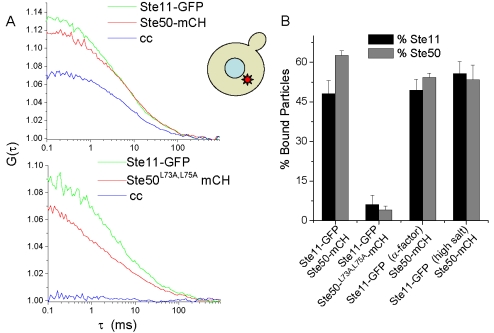
Cross-correlation analysis determines protein co-diffusion. A. Autocorrelation and cross-correlation curves of Ste11-GFP and Ste50-mCherry and Ste11-GFP and Ste50-^L73A-L75A^-mCherry. Curves are the averages of multiple cells. B. Results for G1 cycling cells, and cells upon pheromone or osmotic stress pathway activation in live yeast cells (see Materials and Methods). Results were quantified as previously described, as the percentage of bound particles relative to total [27], [39].

For examining Ste11 and Ste50 interaction, we constructed a yeast strain expressing Ste11-GFP and Ste50-mCherry from their respective chromosomal loci (Table 1). Fluorescence cross-correlation spectroscopy measurements were first performed as previously described [27] to confirm the expected heterotypic interaction (Figure 2). All of the fluorescence fluctuation measurements described in this study were made on the cytosolic pool by appropriately targeting the laser beam (Figure 2A and Materials and Methods). As expected, a high degree of cross-correlation was observed (Figure 2A, 2B), as demonstrated by the high amplitude of the cross-correlation curve relative to the autocorrelation curves of the individual channels. Results were quantified [39] and showed over 60% of Ste50 bound with Ste11 (Figure 2B). The interaction of these proteins remained strong after activation of the pheromone response pathway or the osmotic stress pathway (Figure 2B) (see Materials and Methods). To test if the observed interaction relies on the SAM domain, two mutations, L73A and L75A, were introduced into the Ste50 SAM domain (Table 1). These mutations were previously shown to abolish the binding between Ste11 and Ste50 SAM domains *in vitro* [17]. Ste50^L73A-L75A^-GFP was driven by the *STE50* promoter from a centromeric plasmid and expressed in the *ste50Δ* background. The cross correlation was diminished (Figure 2A, 2B), demonstrating as expected that the Ste50 SAM domain plays a role in Ste50's interaction with Ste11 *in vivo*.

**Table 1:** Yeast strains used in this study.

Strain	Genotype	Source
rly		[27]
2667	MATA *BAT2-GFP-mCherry::URA3* (6 Ala linker)	[27]
2748	MATA p*BZZ1::GFP:HIS5*	[27]
3118	MATA ;*STE11-GFP::HIS5*	[27]
3120	MATA ;*STE50-GFP::HIS5*	this study
3126	MATA p*BZZ1::GFP-GFP-GFP:URA3*	[27]
3165	MATA p*BZZ1::GFP-GFP:URA3*	[27]
3232	MATA ;*STE11-GFP::HIS5 STE50mCherry::URA3*	this study
3282	MATA ;*ste50Δ*; *STE11-GFP::URA3*; CEN *HIS5* plSTE50^L73AL75A^-*mCherry*	this study
3283	MATA ;*ste50Δ*; CEN *HIS5* plSTE50^L73AL75A^-*GFP*	this study
3291	MATA ;p*BZZ1::GFP:HIS5* p*BAT2*:*:mCHERRY::URA3*	this study
3489	MATA p*BZZ1::mCherry-mCherry::URA3*	this study

all strains are S288C background, his3Δ1;leu2Δ0;met15Δ0;ura3Δ0.

**Figure 3:**
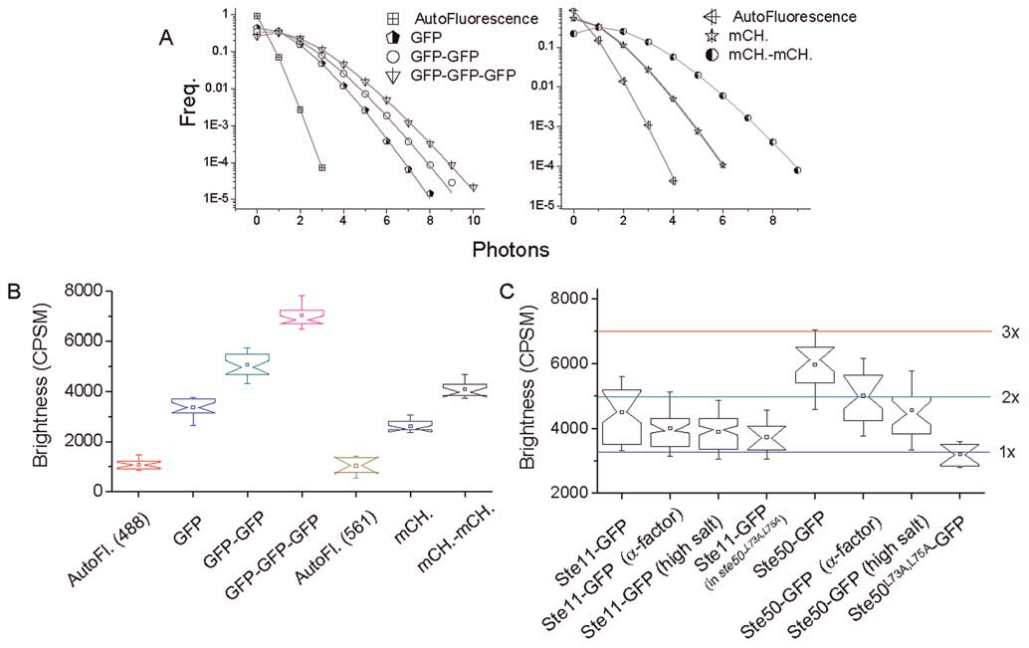
1dPCH analysis of Ste50-GFP and Ste11-GFP probes homo-oligomerization. A. Example curves for GFP and mCherry (mCH.) controls in live yeast. B. Notched box plots of PCH fits, ranging from 18 to 30 individual, 7 second data traces from 5 to 10 cells. For auto-fluorescence measurements, data represents 7 measurements for mCherry and 15 measurements for GFP. 50 µs bins were used to generate the PCH distributions. C. Notched box plots of 1dPCH fits of GFP tagged species, with lines (same color scheme as in B) representing average brightness values of monomer, dimer, and trimer controls for a basis of comparison.

*In vitro*, the Ste11 and Ste50 SAM domains have been shown to mediate homo-oligomerization, but this has not been demonstrated *in vivo* when proteins are expressed at the endogenous levels. PCH analyzes the probability distribution of detected photons from a small confocal volume to calculate particle concentration and brightness, usually reported as the average number of molecules in the focal volume (N) and counts per second per molecule (CPSM), respectively (Figure 1C) [29], [40]. As a comparison technique, molecular brightness reflects the oligomeric state of the fluorescent species when compared to the brightness of a standard, for example, a known monomer or dimer of the same fluorescent molecule. For controls, yeast strains expressing monomeric, dimeric, and trimeric cytosolic GFP under the control of the BZZ1 promoter were constructed, as previously described (Table 1) [27]. The distribution of brightness values for 1dPCH measurements in live yeast cells were recorded using the Zeiss confocor 3 with 488 nm excitation and BP 505–540 nm emission collection (Materials and Methods). Box plots, as well as example curves, are shown in Figure 3A and 3B. The brightness distributions of these GFP species were easily distinguishable, providing the basis of comparison for oligomeric status of mobile GFP-tagged proteins in yeast. In addition, the *BAT2* locus was replaced by cytosolic mCherry and the *BZZ1* locus was replaced by mCherry-mCherry (Table 1). At the low excitation powers that are necessary to minimize photobleaching in our experiments, the brightness of mCherry was less than that of eGFP (Figure 3B) but, at over 2000 CPSM, still presented an improvement from other monomeric, red autofluorescent protein options [35], as expected based on improvements in photostability, and quantum yield [37], [38].

Box plots of average brightness for individual 1dPCH measurements of Ste11 and Ste50 are presented in Figure 3C, with lines representing average brightness values of monomer, dimer, and trimer controls for a basis of comparison. Ste11 exhibited an average brightness close to that of dimeric cytosolic GFP, whereas Ste50 showed an average brightness in-between dimeric and trimeric cytosolic GFP. Interestingly, the SAM domain mutant, Ste50^L73A-L75A^-GFP, revealed a brightness much reduced relative to Ste50-GFP, near that of monomeric GFP, suggesting that these mutations also affect homo-oligomerization of Ste50. Surprisingly, PCH of Ste11-GFP in a yeast strain where the only form of Ste50 was untagged Ste50^L73A-L75A^ revealed a decreased brightness, distinct from the distribution of Ste11-GFP in wild-type cells (p<0.05), suggesting that the SAM domain of Ste50 is also required for stabilization of Ste11 homo-oligomers.

To examine the effect of signals that normally activate MAP kinase cascade on Ste11-GFP and Ste50-GFP complexes, we activated the yeast mating response pathway by treatment of yeast cells with 50 µM α-factor or the osmotic stress pathway by treating the cells with 0.4 mM NaCl for 30 minutes [14] (see Materials and Methods). Average brightness values for PCH measurements are shown in Figure 3C. The average brightness of Ste11-GFP and Ste50-GFP were slightly decreased in response to both conditions.

The average brightness values of Ste11-GFP and Ste50-GFP, which were above the brightness of monomeric GFP, suggested an ability of these proteins to form homo-oligomeric structures, but the composition of the complexes was unclear. This represents a difficulty with 1dPCH. For example, the average brightness of Ste50-GFP could be explained by a distribution of dimers and trimers, but could also be explained by a distribution of monomers and high-order oligomers, or any other combination. In an ideal case, such as a solution measurement, sufficient statistics can be obtained to accurately distinguish a distribution of species freely from PCH data without *a priori* knowledge. However, this is not the case in live yeast cells due to limits of laser exposure time to minimize photobleaching. Similarly, other live cell studies have also found it necessary to make certain reasonable assumptions and/or fix brightness values to fit fluctuation data to distributions to extract additional information [32], [40].

**Figure 4:**
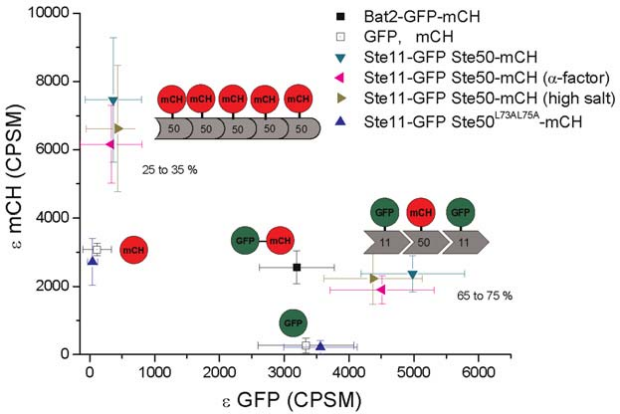
2dPCH analysis of Ste50-mCherry and Ste11-GFP detects binding stoichiometry. 50 µs bins were used. Data were fit to a one-component model or two-component model, as explained in the text. Symbols and bars represent the averages and standard deviations, respectively. Schematic representations of average stoichiometry observed; possible geometries of the interactions (see main text) are displayed next to the corresponding regions of the graph.

To better examine the stoichiometry of the Ste11 and Ste50 complexes, 2dPCH was performed. As a proof of principle, we first applied 2dPCH to a yeast strain expressing GFP and mCherry physically linked to the cytosolic protein Bat2 [27] (Table 1). The 2dPCH histograms of Bat2-GFP-mCherry fit well to a 1 species model, with diffusing particles having coincidence brightness in the GFP and mCherry channels with values consistent with monomeric GFP and mCherry (Figure 4, compare to Figure 3B). As a negative control, 2dPCH was conducted for a yeast strain expressing unlinked, cytosolic GFP and cytosolic mCherry (Table 1). As expected, the data fit well with a two-species model (average chi^2^ = 0.9) but not with a one-specie model (average chi^2^ = 4.5). The resulting brightness values for the 2dPCH data sets were consistent with those expected for monomeric GFP and mCherry. In all two-species 2dPCH fits, an F test was used to validate the necessity of the second-component (F>97%).

2dPCH was applied to the Ste11-GFP, Ste50-mCherry fluctuation data, and revealed the *in vivo* binding stoichiometry of the complexes (Figure 4). Again, a one-specie model did not adequately fit the data (average chi^2^ = 4.1); a two-component fit was necessary (average chi^2^ = 1.2). The data reveal a dominant specie (N comprises approximately 65 to 75% of the total particles from the fit) with a mCherry average brightness (2400±150) consistent with that of a monomer (p = 0.12) and a GFP average brightness of 4980±230, which is indistinguishable from the 1dPCH GFP-GFP dimer brightness (p = 0.7). Thus, the data suggests a dominant complex in the yeast cytosol consisting of monomeric Ste50 and dimeric Ste11. A second abundant specie revealed by the 2dPCH consisted of a high order oligomer of Ste50 that is not associated with Ste11. This data reveals a mutual exclusiveness between Ste50 homo-oligomerization and Ste50 forming a complex that contains two molecules of Ste11. Based on 1dPCH data, we expected a small fraction of a third specie, consisting of monomeric Ste11, but a three-component fit cannot be confirmed with statistics provided by the live cell measurements. Consistent with the lack of cross-correlation reported in Figure 2, 2dPCH of Ste11-GFP, Ste50^L73A-L75A^-mCherry did not fit with a one-specie model (average chi^2^ = 6.8), but rather a two-species model (average chi^2^ = 1.2), with non-interacting, monomeric species. This result confirms the FCCS and 1dPCH results that the Ste50 SAM domain is required for homo-oligomerization of Ste50, interaction of Ste50 with Ste11, and it plays a role in stabilization of the Ste11 dimer.

The effects of activation of the mating pathway and osmotic stress pathway were subtle, with the dominant specie in either case still consisting of monomeric Ste50 interacting with Ste11. The average GFP brightness value of this dominant complex under these conditions were lower than that in cycling cells; this decrease was statistically significant at the 95% level for high salt conditions relative to wt (p = 0.05) but not at the 95% confidence limit for α-factor treated cells (p = 0.11). The trend is consistent with a lower average brightness of the Ste11 component of this complex, and perhaps a distribution of interacting species that varies between 2∶1 and 1∶1 Ste11∶Ste50. The average brightness of the Ste50 high-order oligomer observed by 2dPCH was significantly reduced upon activation of the pheromone pathway (p = 0.02), while the average brightness of the oligomer upon activation of the osmotic stress pathway was not reduced at a statistically high confidence level (p = 0.24).

**Figure 5:**
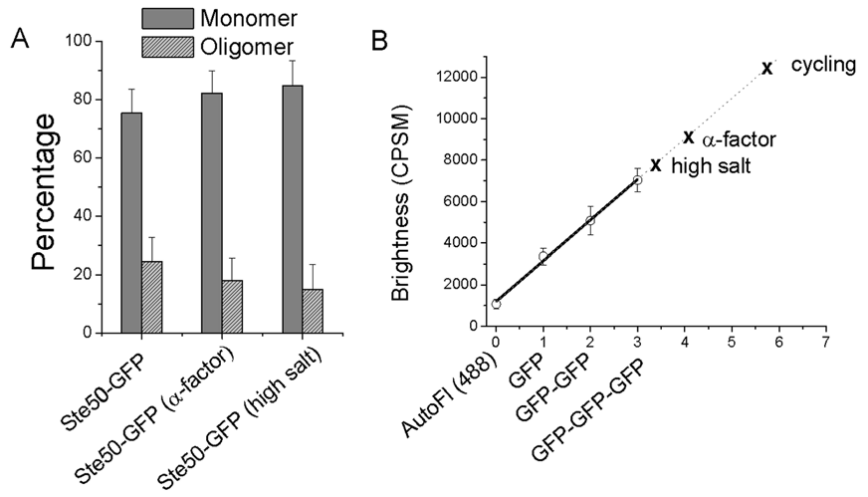
Constrained fits of the 1dPCH data to two-species allows for the examination of monomer and oligomer populations. A. A model was assumed where Ste50 could exist as either a monomer with fixed brightness, or oligomer with unconstrained brightness and number (see Materials and Methods, and main text). Results are displayed to show the percentage of each component. Error bars are the standard error of the mean. B. Average brightness values for autofluorescence, GFP, GFP-GFP, and GFP-GFP-GFP from the data in Figure 3, fit to a line. Error bars are the standard deviation. The line represents the best fit of the data to a linear model with a slope of 1959 and intercept 1193, which was then extrapolated toward higher brightness. Average brightness observed for the Ste50 oligomer from the analysis described above are marked on the extrapolated part of the plot.

Thus, a possible effect of activation of these pathways is a trend toward a reduction in the size of the high-order Ste50 oligomer. This is consistent with change observed by 1dPCH as the decreased average brightness of Ste50-GFP upon activation of the signaling pathways. However, at this point we are uncertain how brightness of mCherry containing complexes scales with number of mCherry subunits at high stoichiometry. The brightness of GFP complexes scales well with GFP subunits, as demonstrated by the fit of the average brightness of the monomeric, dimer, and trimeric controls (Figure 5). We revisited the 1dPCH data of Ste50-GFP to attempt to better quantify the stoichiometry of the high-order, Ste50 oligomer. The result that the dominant specie of Ste50 was a monomer, as shown by 2dPCH, provided an important constraint for fitting the 1dPCH data to a distribution. Therefore the 1dPCH data for Ste50-GFP was fit to a distribution consisting of a fixed monomer brightness and freely varied oligomer brightness. N for each species was also freely varied. The Ste50 1dPCH data was well fit (average chi^2^ = 0.9) to a distribution that consists of a large percentage of monomer and a small percentage of high order oligomer, in percentages roughly equivalent with those found using 2dPCH. The constrained fits revealed that the treatment with α-factor or high salt led to a slightly decreased percentage of oligomer, and also a decreased oligomer brightness (Figure 5). Using this analysis, average brightness values of the Ste50 oligomer were approximately 12,500, 8900, and 7700 CPSM for cycling cells, α-factor treated cells, and cells at high salt, respectively (Figure 5). Assuming that the linearity of GFP brightness continues to hold at a high number of subunits, we estimate the number of Ste50 subunits in this complex to be ∼5 to 6 in cycling cells, ∼4 in α-factor treated cells, and ∼3 to 4 in high salt condition. While the slight decrease in brightness of the Ste50 oligomer was also observed with mCherry in the 2dPCH data, it was not nearly as pronounced as that observed with 1dPCH (Figure 3D) or the global fit (Figure 5), raising the possibility that mCherry brightness may not scale linearly with subunit number at high stoichiometry, perhaps due to a self-quenching mechanism or increased propensity for photobleaching or photoblinking relative to GFP.

## Discussion

The analyses presented above demonstrate that whereas FCCS and 1dPCH provide valuable information on the strength of the interaction between two molecular species and average oligomerization status of individual molecular species, respectively, 2dPCH is better suited for revealing the binding stoichiometry of protein complexes *in vivo.* Specifically in this study, 2dPCH revealed a predominant complex of Ste11 and Ste50 with a 2∶1 binding stoichiometry, and a pool of large, Ste11-free Ste50 oligomers. The heterotypic complex is consistent with previous biochemical data [17], [18], [21]. However, the requirement of Ste50 SAM domain for the integrity of this complex *in vivo* suggests that either Ste11 molecules in this complex do not directly dimerize through their SAM domains but rather they each bind a common Ste50 SAM domain at two different surfaces (Figure 4); or, a strong direct interaction between two Ste11 molecules is stabilized by the Ste50 SAM domain. The former possibility is supported by the structural study demonstrating that Ste50 binds Ste11 in a head to tail fashion [19], which could also explain the ability of Ste50 to form homo-oligomers (Figure 4). The most unexpected finding was the presence of large Ste50, but not Ste11, homo-oligomers, because *in vitro* the Ste11 SAM domain, but not the Ste50 SAM domain, has the strong propensity to form oligomers [2], [6], [20], [21]. Even though we do not have an explanation for the difference between the *in vivo* and *in vitro* observations, this result highlights the need to directly probe protein complex stoichiometry using techniques such as 2dPCH in live-cell settings.

The SAM domain-mediated interaction between Ste50 and Ste11 is known to be important for efficient MAP kinase signaling during mating and osmotic stress responses [12], [15]. It is thought that this interaction facilitates the targeting of Ste11 to the site of receptor signaling at the plasma membrane where Ste20, the upstream kinase for Ste11, is activated by the small GTPase Cdc42 [12], [14], [15], [41] Ste50 itself is recruited to the membrane through an interaction of the C-terminal RA domain with Cdc42 [16]. An ability of one Ste50 to simultaneously bring two molecules of Ste11 to the site of active Ste20 on the cortex may significantly enhance the efficiency of Ste11 phosphorylation by Ste20, while the large Ste50 homo-oligomers may be a dynamic reservoir for Ste50 that could buffer the concentration of Ste50 monomers available for interaction with Ste11. A recent study showed that the mobile Ste11 concentration increases following pheromone stimulation [27], and this would be consistent with a need to mobilize some of the Ste50 reserved in the homo-oligomers to the monomer pool, leading to the reduced average size of the Ste50 oligomer.

The homo-oligomers of Ste50 observed in yeast may be similar to the high affinity, high-order oligomers proteins observed for SAM domains from proteins such as translocation ETS leukemia (TEL) or Eph receptor tyrosine kinase (EphB2) [6], [42]. As mentioned above, the constrained two-component fit of the 1dPCH data for Ste50-GFP reported a stoichiometry of the Ste50-oligomer in cycling cells of 5 to 6 subunits. Given the caveat that the brightness of GFP might not rise linearly with high-order oligomers, it is still intriguing that the estimated stoichiometry of Ste50 *in vivo* compares well to the stoichiometry proposed for oligomers of TEL or EphB2 [2], [6], [42]. While these models generally predict a linear or head-to-tail model, if this applies to the Ste50 oligomer it would be difficult to explain the observed lack of binding to Ste11. One possibility is that formation of the Ste50 oligomer induces a conformational change in the Ste50 SAM domain that makes binding to Ste11 less likely, as depicted in Figure 4. It may be interesting to test if the TEL SAM domain homo-oligomers, which are thought to account for certain types of human leukemia [2], [6], [9]–[11], also modulate the interaction with other regulatory molecules and respond dynamically to morphogenetic signals.

## Materials and Methods

### Yeast culture

One-step COOH-terminus genomic tagging was used for generating yeast strains expressing both GFP and mCherry labeled genes [43], [44] unless otherwise specified. Correct tagging was verified by PCR. For correlation analysis, yeast cells were grown in synthetic complete media to mid-log phase. For examination of Ste50 SAM mutants, Ste50-GFP or Ste50-mCherry was subcloned into a centromeric plasmid, with its promoter region. Mutations were made and verified with sequencing. The centromeric plasmids were transformed into *Δste50* strains for analysis. For examination of the mating pathway, pheromone (α-factor) was incubated with yeast at a concentration of 50 µM for 2 hours. For activation of the HOG pathway, yeast cells were treated with 0.4 mM NaCl for 30 minutes as described [14]. Yeast cells were immobilized on glass for analysis.

### FCS

The experimental set-up for FCS, FCCS, and 1dPCH was used as previously described [27]. Briefly, for cross-correlation studies, the 488 nm and 561 nm laser lines were used with the HFT 488/561 dichroic to excite GFP and mCherry, respectively. Emission was split with an HFT565 dichroic, and a 505–540 BP emission filter was used for the green emission channel and a LP 580 nm filter for the red channel. The pinhole was set to 1.0 airy unit in the red channel. Autocorrelation curves for the individual channels and a cross-correlation curve between the channels was calculated by the Zeiss Confocor-3 software according to equations 1 and 2, respectively:
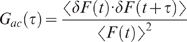
(1)

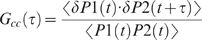
(2)where *δF(t) = F(t)−〈F〉* and *P1* and *P2* represent photon counts in channel 1 and 2, respectively.

For cross-correlation experiments, the number of bound particles was calculated from Eq. 3 [39], where the inverse amplitude of the autocorrelation curves was used to calculate the number of red (*N_RT_*) or green particles (*N_GT_*) (Eq. 4). *N_cc_* is the inverse amplitude of the cross-correlation curve, and the cross-talk between channels, *Q*, was estimated to be approximately 5% for the GFP and mCherry probes using the filter sets described above. This is discussed in more detail elsewhere [27]. A volume correction was applied to take into account small differences in red, green, and cross-correlation volumes [45].
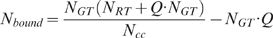
(3)

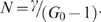
(4)


### PCH

1dPCH was conducted as previously described [27]. The 488 nm laser line was used with a HFT 488/561 main dichroic, an HFT565 secondary emission dichroic, and a 505–540 BP emission filter. Fluorescence traces were collected in 7 second increments, and 4 to 5 measurements were collected per cell. Data was arranged as a histogram of number of photon events per unit time using a bin time of 50 µs. Data was fit with the PCH algorithm [29], [30] to extract an average brightness and particle number per measurement. A 3-dimensional Gaussian focal volume (1-photon) was used. Control strains expressing GFP, GFP-GFP and GFP-GFP-GFP linked proteins (under the *BZZ1* promoter) were used for monomer, dimer, and trimer brightness controls (Table 1). Importantly, to verify the reliability of 1-photon PCH in live yeast, the brightness values for the control strains were linearly spaced, taking auto-fluorescence into account.

For 2dPCH [35], [36], the experimental set-up was identical to the cross-correlation set-up described above and previously [27]. Data were taken in ten-second increments, and arranged in a two-dimensional histogram of counts in each channel as a function of frequency for 50 µs bins. Data were fit to one or two components, and brightness values and particle number in each channel were allowed to freely vary. An F-test was used to validate the necessity of two-component fits (F>97%). The plots in Figure 4 represent averages and standard deviations for N between 6 and 15 cells.

For fitting Ste50-GFP, 1dPCH data to a two-component model, as shown in Figure 5, the brightness of a monomer specie was fixed. The number of monomer, brightness of oligomer, and number of oligomer were freely varied.
